# Can artificial intelligence pass the Fellowship of the Royal College of Radiologists examination? Multi-reader diagnostic accuracy study

**DOI:** 10.1136/bmj-2022-072826

**Published:** 2022-12-21

**Authors:** Susan Cheng Shelmerdine, Helena Martin, Kapil Shirodkar, Sameer Shamshuddin, Jonathan Richard Weir-McCall, FRCR-AI Study Collaborators, Sayed Hashim AlQarooni, Sajay Alias, Saraswati Samyukta Aryasomayajula, Caline Azzi, Awadh Ahmed Taiseir Ba Hashwan, Anintitha Boon-Itt, Momana Tariq Butt, Liisa Chang, Victoria Crowe, Mahmoud Mohamed Elsaidy, Aejaz Ahmed Gonegandla, Abeeku Afedzi Hammond, Mansi Jantre, Kavya Moonjelil Karthikeyan, Sharon Koo, Sophie McGlade, Aprajita Mehta, Preeti Mundhada, Marwa Nayel, Wee Ping Ngu, Ryan Norman, Amina Odeh, Dileep Kumar Perumala, Shayeri Roy Choudhury, Sarath Babu Stephen Maria Lourdam, Anil Kumar Geetha Virupakshappa

**Affiliations:** 1 Department of Clinical Radiology, Great Ormond Street Hospital for Children, London, UK; 2 UCL Great Ormond Street Institute of Child Health, Great Ormond Street Hospital for Children, London, UK; 3 NIHR Great Ormond Street Hospital Biomedical Research Centre, London, UK; 4 Department of Clinical Radiology, St George’s Hospital, London, UK; 5 Department of Radiology, University Hospitals of Morecambe Bay NHS Trust, Royal Lancaster Infirmary, Lancaster, UK; 6 School of Clinical Medicine, University of Cambridge, Cambridge, UK; 7 Department of Radiology, Royal Papworth Hospital, Cambridge, UK

## Abstract

**Objective:**

To determine whether an artificial intelligence candidate could pass the rapid (radiographic) reporting component of the Fellowship of the Royal College of Radiologists (FRCR) examination.

**Design:**

Prospective multi-reader diagnostic accuracy study.

**Setting:**

United Kingdom.

**Participants:**

One artificial intelligence candidate (Smarturgences, Milvue) and 26 radiologists who had passed the FRCR examination in the preceding 12 months.

**Main outcome measures:**

Accuracy and pass rate of the artificial intelligence compared with radiologists across 10 mock FRCR rapid reporting examinations (each examination containing 30 radiographs, requiring 90% accuracy rate to pass).

**Results:**

When non-interpretable images were excluded from the analysis, the artificial intelligence candidate achieved an average overall accuracy of 79.5% (95% confidence interval 74.1% to 84.3%) and passed two of 10 mock FRCR examinations. The average radiologist achieved an average accuracy of 84.8% (76.1-91.9%) and passed four of 10 mock examinations. The sensitivity for the artificial intelligence was 83.6% (95% confidence interval 76.2% to 89.4%) and the specificity was 75.2% (66.7% to 82.5%), compared with summary estimates across all radiologists of 84.1% (81.0% to 87.0%) and 87.3% (85.0% to 89.3%). Across 148/300 radiographs that were correctly interpreted by >90% of radiologists, the artificial intelligence candidate was incorrect in 14/148 (9%). In 20/300 radiographs that most (>50%) radiologists interpreted incorrectly, the artificial intelligence candidate was correct in 10/20 (50%). Most imaging pitfalls related to interpretation of musculoskeletal rather than chest radiographs.

**Conclusions:**

When special dispensation for the artificial intelligence candidate was provided (that is, exclusion of non-interpretable images), the artificial intelligence candidate was able to pass two of 10 mock examinations. Potential exists for the artificial intelligence candidate to improve its radiographic interpretation skills by focusing on musculoskeletal cases and learning to interpret radiographs of the axial skeleton and abdomen that are currently considered “non-interpretable.”

## Introduction

In 2016 Geoffrey Hinton, winner of the Turing award and considered one of the godfathers of deep learning, proclaimed: “We should stop training radiologists now. It’s just completely obvious that within five years, deep learning is going to do better than radiologists.”^1^ We are now five years past this seminal statement, so the time is ripe to put artificial intelligence to the test and see if it is ready to graduate.

Radiologists in the UK are required to pass the Fellowship of the Royal College of Radiologists (FRCR) examination before their completion of training, which allows them to practice independently as radiology consultants.^2^ ^3^ For artificial intelligence to replace radiologists, ensuring that it too can pass the same examination would seem prudent. Three components make up the final FRCR examination, for all of which candidates need a pass mark to pass the full examination overall. One of these three components is called the “rapid reporting” session. In this session, candidates must interpret 30 radiographs within 35 minutes. The candidate must correctly report at least 27 (90%) of these 30 radiographs to pass this component.^4^ ^5^ This part of the examination is designed to “stress test” candidates for speed and accuracy, providing a mixture of challenging normal and abnormal cases typically referred by general practice and the emergency department for radiological interpretation in clinical practice. Speed, accuracy, binary outcomes, and radiographs are all areas in which artificial intelligence has been purported to excel,^6^ ^7^ so the rapid reporting component of the FRCR examination should be an ideal test setting in which to evaluate its prowess.

Imagine that today is examination day. The artificial intelligence algorithm has been receiving extensive training, reviewing thousands of radiographs and receiving feedback to hone its diagnostic acumen. Alongside several of its human peers, it will take the FRCR rapid reporting examinations and see whether it can come one step closer to obtaining a diploma from the Royal College of Radiologists (RCR). The aim of this UK based multicentre, multi-reader prospective comparative study was therefore to determine how well an “artificial intelligence candidate” would perform across a series of FRCR rapid reporting examinations and whether it might outperform some of its human (radiologist) peers.

## Methods

### FRCR mock examinations

We approached the RCR and asked for “retired” FRCR rapid reporting examination cases to be shared to allow for an accurate representation of the actual examination. Unfortunately, this request was denied owing to a desire to protect the integrity of the FRCR examination (personal email communication).

As an alternative, we used 10 FRCR mock rapid reporting examinations for analysis. The lead author had previously created these and used them over the preceding five years as revision material for radiology trainees at the local institution. The radiographs were selected to reflect the same or a higher level of difficulty and breadth of knowledge expected for the real FRCR examination. The cases had not been used for any national or international training or revision courses. All imaging results from the mock examinations had previously been verified and reviewed by two consultant radiologists and with follow-up imaging (where available) to ensure the accuracy of the imaging findings (that is, our ground truth).

As per the official FRCR rapid reporting examination component, each mock examination consisted of 30 radiographs (some with multiple projections/views), with approximately half containing no abnormalities and the rest containing only one pathology (multiple lung nodules are counted as the same single pathology).^5^ The radiographs covered all body parts and were a mixture of images from adult and paediatric patients (supplementary tables A and B). The RCR sets no pre-specified split of body parts or paediatric cases per examination. Although the actual number of abnormal radiographs is unknown to candidates sitting the real FRCR rapid reporting examination, they know beforehand that this will be approximately half (40-60%) of the radiographs.^5^ Clinical information is not provided to candidates in the rapid reporting component.

### Radiologist readers

We recruited human participants (that is, “radiologist readers”) via email, social media, and word of mouth from previous successful FRCR candidates from the emailing list of a popular international FRCR examination revision course (www.frcrtutorials.com). None of the imaging cases included in this study had been previously shown to the participants on this revision course.

We included radiologists who had passed their FRCR examination within the previous 12 months, to represent the appropriate level and experience of a recently “FRCR qualified” trainee. Radiologists who were recent trainees at the lead author’s institute (and thus could have potentially seen the mock examinations as part of their examination revision) were excluded from participation. We asked all readers to complete a consent form before participation and a short survey outlining number of previous FRCR examination attempts and demographic details on age and gender.

### Human (radiologist) image interpretation

We provided the anonymised radiographic images via a secure, password protected, and General Data Protection Regulation compliant online “digital imaging and communications in medicine” (DICOM) image viewing platform (Collective Minds Radiology; https://www.cmrad.com/). Each radiographic image could be manipulated by the study participants on this platform in the same way as allowed in the real examination (that is, changing the image’s brightness, orientation, and rotation and increasing its magnification).

We asked radiologists to note their interpretations (that is, normal or abnormal, and if abnormal then what pathology) on an online data collection sheet for each mock examination. The participants interpreted the images remotely at their own convenience, but we asked them to do all readings under timed (35 minutes) examination conditions in a quiet, undisturbed location and using a suitable computer screen monitor with dim lighting. We gave radiologists one month to provide their imaging interpretations for the 10 mock examinations (1 May to 31 May 2022).

At the end of each mock examination, we asked participants to rate on a 10 point Likert-type scale how representative they thought each of the 10 mock examinations was of the actual FRCR rapid reporting component, how well they thought they had performed, and how well they believed a commercially available artificial intelligence tool would have performed.

### Artificial intelligence analysis

We also provided all 300 anonymised radiographs across the 10 mock FRCR examinations to the artificial intelligence candidate in an anonymised DICOM format. DICOM files are the primary file format for storing and transferring medical images in hospital imaging databases. They contain information about the image (called metadata) that specifies parameters for how the image was acquired. Using a DICOM file format ensures that underlying information about an image is not lost during image transfer/sharing as it supports “lossless” decompression, unlike other file formats such as jpeg.

The artificial intelligence candidate was a commercially available tool called Smarturgences v1.17.0, developed by a French artificial intelligence company called Milvue (https://www.milvue.com/english/home) and marketed since February 2020. The tool has been awarded Conformitè Europëenne certification under the Medical Devices Directive and registered as a class 2a medical device.^8^ It is used in more than 10 institutions across Europe as part of clinical care, although not currently in the UK. The artificial intelligence model had been trained on a multicentric dataset of more than 600 000 chest and musculoskeletal radiographs to detect seven key pathologies (fracture, pleural effusion, lung opacification, joint effusion, lung nodules, pneumothorax, and joint dislocation) by displaying a bounding box on the radiograph corresponding to the area of abnormality, with output descriptors provided in French. For each positive finding the artificial intelligence tool also provides a binary certainty score (that is, certain/positive or uncertain/doubtful). For the purposes of this study, all positive findings, regardless of the assigned certainty, were considered the same.

We chose this tool as our artificial intelligence candidate because it was the only commercial product able to analyse both musculoskeletal and chest radiographs (other products were able to do only one of these tasks). Although it is not certified to analyse radiographs of the axial skeleton (that is, skull, spine, and dental views) or abdominal radiographs, we still provided radiographs pertaining to these body parts across the 10 mock rapid reporting examinations for artificial intelligence analysis to maintain examination fairness across all participants.

We assigned analyses provided by the artificial intelligence tool that correlated with our ground truth as true positives or negatives, those for which the abnormality was not identified as false negatives, and normal radiographs for which an abnormality was assigned by the artificial intelligence as false positives. Where more than one abnormality was identified, we judged the artificial intelligence tool result to be wrong (false positive), as none of the radiographs depicted more than one pathological process. This is similar to how a radiology candidate would be scored in the real FRCR if they were to provide several abnormalities.

### Statistical analysis

#### Human participants

We calculated the mean, median, and range of examination scores (with percentages) across each of the 10 mock examinations for all radiologist participants. We assigned a pass mark of 27/30 (90%), in line with the scoring criteria used by the RCR. We also calculated the sensitivity, specificity, and positive and negative predictive values per radiologist. In addition, we calculated the mean, median, and range of scores for radiologists’ perceptions of how representative the mock examinations were of the actual FRCR rapid reporting examination, how well they believed they had performed, and how well they believed the artificial intelligence model would perform.

#### Commercial artificial intelligence tool

Given that some of the radiographs in each of the rapid reporting examinations would be uninterpretable by the artificial intelligence tool (for example, axial skeleton, facial bones), we calculated the examination score for the artificial intelligence in four different ways.

*Scenario 1*—scoring only the radiographs the artificial intelligence model could interpret. In this scenario, we excluded any radiographs the artificial intelligence model deemed “non-interpretable.” A score for each mock examination was based on only those radiographs that could be interpreted (therefore, total marks per examination could be less than the available 30, depending on the number of non-interpretable radiographs per set). This scenario would be akin to a generous examiner making exceptions for the candidate.

*Scenario 2*—scoring all non-interpretable radiographs as “normal.” In this scenario, we imagined that the “artificial intelligence candidate” had not prepared sufficiently for the examination and could not interpret certain radiographs. Given the lack of negative marking in the examination, we imagined that the artificial intelligence candidate took a chance and assigned a default answer of “normal” for each non-interpretable case as this would be better than leaving it blank. We assigned a total score out of 30 marks. Abnormal non-interpretable cases were therefore calculated as false negatives, and normal non-interpretable cases were calculated as true negatives.

*Scenario 3*—scoring all non-interpretable radiographs as “abnormal.” In this scenario, we imagined that the “artificial intelligence candidate” attempted the opposite tactic to scenario 2 and assigned a default answer of “abnormal” for each non-interpretable case. We assumed that where an abnormality was present it was correct. We assigned a total score out of 30 marks. Abnormal non-interpretable cases were therefore calculated as true positives, but normal non-interpretable cases were calculated as false positives.

*Scenario 4*—Scoring all non-interpretable radiographs as wrong. In this scenario, the “artificial intelligence candidate” had simply chosen not to commit to an answer and left the answer box blank for non-interpretable cases. Therefore, the total score for each examination was out of 30, and we assigned no marks to non-interpretable radiographs (as would be the case for a human radiologist in the real examination). This therefore represents the most realistic like-for-like marking method in real life. For the purposes of the confusion matrix, we assumed that all non-interpretable radiographs were “wrong” and calculated those that were abnormal as false negatives and those that were normal as false positives.

For ease of comparison between the radiologists’ performance and that of the artificial intelligence, we pooled results for summation of the accuracy of the radiologists across all 10 reporting sets (300 films in total, and also for the subset that the artificial intelligence could interpret) by using the lme4 package within R (R version 3.6.2^9^) within the RStudio environment (version 1.1.463) to do a bivariate binomial random effects meta-analysis.^10^ This uses a binary (logit) generalised linear mixed model fit by maximum likelihood (using a Laplace approximation). We constructed bivariate summary receiver operator characteristic curves by using the bivariate random effects model outputs. On this summary receiver operator characteristic curve, we superimposed the artificial intelligence global accuracy across the subset of artificial intelligence interpretable radiographs (that is, scenario 1) for comparison.

#### Imaging pitfalls

To understand how and where the artificial intelligence tool could aid or mislead healthcare professionals, we reviewed all cases in which: apparently non-interpretable images were given a diagnosis by the artificial intelligence tool (that is, cases in which the artificial intelligence should have recognised the image was inappropriate for analysis but analysed it erroneously anyway); fewer than 50% of the radiologists could correctly analyse the imaging, and how often the artificial intelligence tool was correct (that is, in which cases artificial intelligence could help radiologists to get “off the fence”); and almost all (>90%) radiologists correctly identified the imaging findings but the artificial intelligence tool was incorrect (that is, abnormalities for which the artificial intelligence may potentially mislead non-radiologists).

### Patient and public involvement

The topic of using artificial intelligence for imaging interpretation in an acute setting has previously been discussed in June 2020 and October 2021 with two PPI steering groups (Great Ormond Street Hospital Young Persons Advisory Group and Parent and Carers Advisory Group). These groups are made up of approximately 30 children and young adults and 15 parents and carers. Feedback from both groups relating to use of artificial intelligence for interpretation of radiographic imaging was largely positive, particularly where diagnostic accuracy rates for the artificial intelligence tool could be shown to be as high as those of specialist radiologists, although autonomous usage of artificial intelligence was highlighted as a major concern requiring a greater level of evidence. Patients and the public welcomed any safe method that could provide accurate and faster results to guide their future treatment or discharge.

## Results

### Demographics of radiologist readers

Twenty six radiologists were recruited and successfully completed all 10 mock FRCR rapid reporting examinations within the designated study period. Of these, 16/26 (62%) were female and most (19/26; 73%) were aged between 31 and 40 years. Most (16/26; 62%) had just passed their FRCR examination within the previous three months, 8/26 (31%) had passed within the previous six months, and 2/26 (8%) had passed within the previous 12 months. More than half of the participants (15/26; 58%) had successfully passed the FRCR examination on their first sitting, 9/26 (35%) had passed on their second sitting, and 2/26 (8%) had passed on their third sitting.

### Performance of artificial intelligence candidate

We provide the confusion matrix for the performance of the artificial intelligence candidate across each mock examination (table 1) and across the different radiographs of body parts (supplementary table C). Table 2 shows the accuracy rates for the performance of the artificial intelligence candidate across the four different examination marking scenarios; a detailed breakdown of the full diagnostic accuracy results for the artificial intelligence candidate across the different scenarios is provided in supplementary table D**.** The individual diagnostic accuracy rates (sensitivity, specificity, positive predictive value, negative predictive value) for each of the 26 radiologists and their summary estimates are provided in supplementary tables E (scenario 1 analysis) and F (scenario 4 analysis).

**Table 1:** Confusion matrix for performance of commercial artificial intelligence model across 10 mock examinations

Examination set	Not interpretable				True positive				False positive			True negative	False negative
	Normal	Abnormal	Total		Certain	Uncertain	Total		Certain	Uncertain	Total		
1	2	1	3		8	2	10		0	2	2	12	3
2	4	3	7		6	2	8		1	3	4	8	3
3	3	2	5		11	3	14		2	3	5	5	1
4	4	2	6		10	2	12		0	3	3	6	3
5	3	1	4		8	3	11		4	3	7	4	4
6	3	0	3		11	0	11		3	0	3	11	2
7	2	2	4		9	1	10		1	1	2	12	2
8	0	3	3		8	3	11		0	1	1	15	0
9	1	0	1		11	0	11		1	2	3	12	3
10	4	1	5		10	4	14		0	1	1	9	1
Total	26	15	41		92	20	112		12	19	31	94	22

Commercial artificial intelligence tool assigned either “certain” or “uncertain” label to positive findings, as shown. For calculating diagnostic accuracy rates, both uncertain and certain findings were treated as positive cases and combined for total scores of true positive or false positives. They are provided here for full granularity.

For calculation of examination diagnostic accuracy rates: in scenario 1, all non-interpretable radiographs were excluded from analysis; in scenario 2, all non-interpretable radiographs were assumed to be normal (ie, normal=true negative; abnormal=false negative); in scenario 3, all non-interpretable radiographs were assumed to be abnormal (ie, normal=false positive; abnormal=true positive); in scenario 4, all non-interpretable radiographs were considered wrongly interpreted (ie, normal=false negative; abnormal=false positive).

**Table 2:** Examination performance of “artificial intelligence (AI) candidate” across four different marking scenarios versus average radiologist for assessment of all 300 images

Examination set	AI candidate accuracy rate, % (95% CI)					Radiologist readers’ accuracy score, % (n=26)				No (%) radiologists passing per examination	AI ranking (scenario 1) out of 27 candidates*
	Scenario 1	Scenario 2	Scenario 3	Scenario 4		Mean	Median	Range	SD		
**1**	81.5 (61.9 to 93.7)	80.0 (61.4 to 92.3)	76.7 (57.7 to 90.1)	71.4 (41.9 to 91.6)		87.2	88.3	76.7-96.7	5.0	13 (50)	23
**2**	69.6 (47.1 to 86.8)	66.7 (47.2 to 82.7)	63.3 (43.9 to 80.1)	57.1 (28.9 to 82.3)		83.8	83.3	63.3-100.0	7.8	7 (27)	26
**3**	76.0 (54.9 to 90.6)	73.3 (54.1 to 87.7)	70.0 (50.6 to 85.3)	82.4 (56.6 to 96.2)		90.6	93.3	73.3-100.0	6.0	21 (81)	26
**4**	75.0 (53.3 to 90.2)	73.3 (54.1 to 87.7)	66.7 (47.2 to 82.7)	70.6 (44.0 to 89.7)		76.9	76.7	66.7-86.7	6.2	0	18
**5**	57.7 (36.9 to 76.7)	60.0 (40.6 to 77.3)	53.3 (34.3 to 71.7)	68.8 (41.3 to 89.0)		77.8	78.3	53.3-100.0	10.7	4 (15)	26
**6**	81.5 (61.9 to 93.7)	83.3 (65.3 to 94.4)	73.3 (54.1 to 87.7)	84.6 (54.6 to 98.1)		84.4	85.0	66.7-93.3	6.9	7 (27)	18
**7**	84.6 (65.1 to 95.6)	80.0 (61.4 to 92.3)	80.0 (61.4 to 92.3)	71.4 (41.9 to 91.6)		88.7	90.0	73.3-96.7	4.8	14 (54)	24
**8**	96.3† (81.0 to 99.9)	86.7† (69.3 to 96.2)	96.7† (82.8 to 99.9)	78.6 (49.2 to 95.3)		84.6	83.3	73.3-93.3	4.6	6 (23)	1
**9**	79.3 (60.3 to 92.0)	80.0 (61.4 to 92.3)	76.7 (57.7 to 90.1)	78.6 (49.2 to 95.3)		89.9	90.0	76.7-96.7	5.3	21 (81)	25
**10**	92.0† (73.9 to 99.0)	90.0† (73.5 to 97.9)	80.0 (61.4 to 92.3)	87.5 (61.7 to 98.5)		88.1	90.0	66.7-96.7	6.9	14 (54)	9
**All examinations**	79.5 (74.1 to 84.3)	77.3 (72.2 to 81.9)	73.7 (68.3 to 78.6)	68.7 (63.1 to 73.9)		85.2	85.8	75.0-91.7	3.9	3 (12)	26

CI=confidence interval; SD=standard deviation.

90% accuracy is taken as pass mark.

Scenario 1: only interpretable radiographs scored; scenario 2: non-interpretable radiographs assigned as “normal”; scenario 3: non-interpretable radiographs assigned as “abnormal”; scenario 4: non-interpretable radiographs assigned as wrong.

* AI ranking (out of 27 candidates: 26 radiologists, 1 AI), assuming marks assigned as per scenario 1.

† AI model performed better than average radiologist (see exam set 8 and 10).

**Fig 1:**
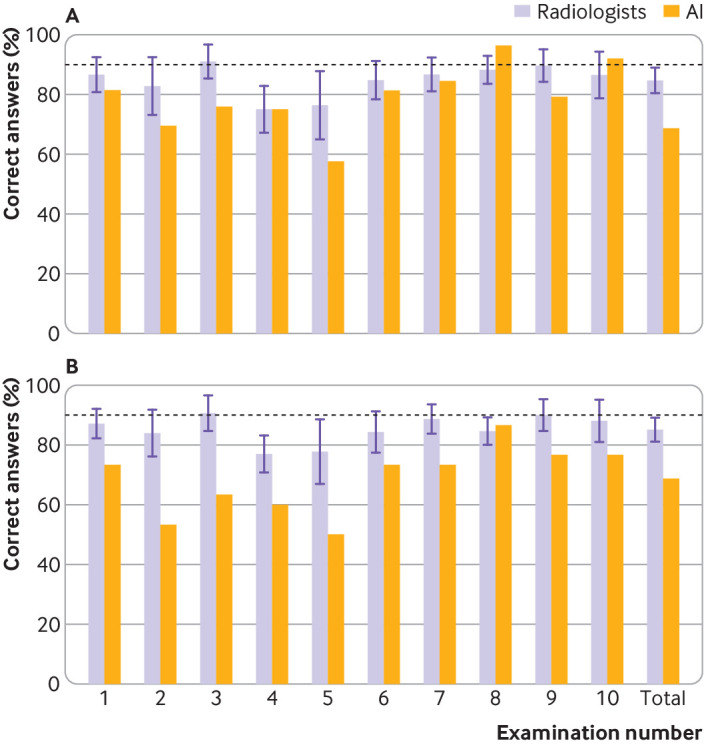
Bar charts showing examination percentage scores per Fellowship of the Royal College of Radiologists mock examination, and overall, acquired by artificial intelligence (AI) candidate and radiologist participants in scenario 1 for only “AI interpretable” images (top) and scenario 4 for all images (bottom). Whisker plots denote standard deviation of scores around mean value by all 26 radiologist participants

In scenario 1 (only “artificial intelligence interpretable” radiographs included), the artificial intelligence candidate would have passed two of 10 mock examinations. In scenario 2 (non-interpretable radiographs assigned as normal), the artificial intelligence candidate would have passed one of 10 mock examinations. In scenario 3 (non-interpretable radiographs assigned as abnormal) and scenario 4 (non-interpretable radiographs assigned as wrong), the artificial intelligence candidate would not have passed any mock examinations (fig 1).

**Fig 2:**
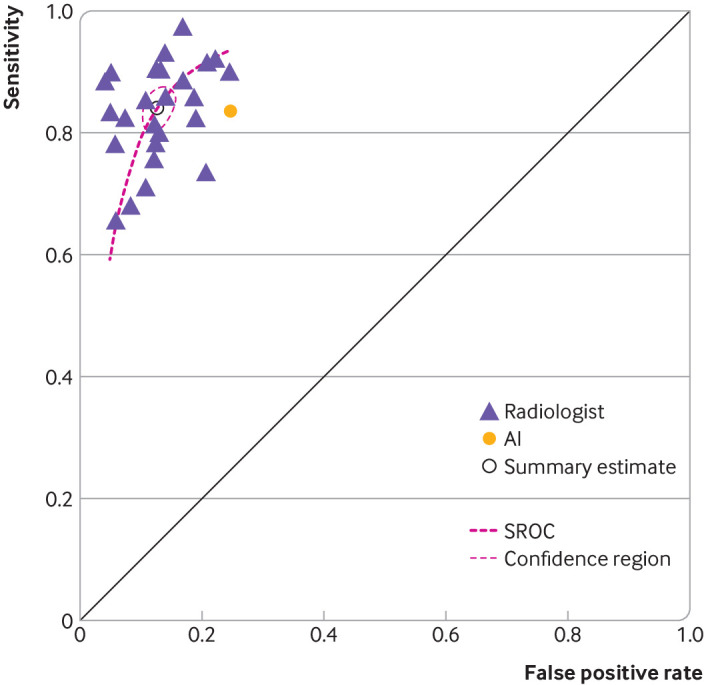
Plot of individual sensitivity and false positive rates of 26 radiologists and artificial intelligence (AI), based on scenario 1 (only “AI interpretable” images) considered. Bivariate random effects summary receiver operator characteristic (SROC) curve and summary estimate for radiologists are included for comparison with AI candidate

When we evaluated only the images the artificial intelligence candidate could interpret (scenario 1), the overall sensitivity for artificial intelligence was 83.6% (95% confidence interval 76.2% to 89.4%), the specificity was 75.2% (66.7% to 82.5%), and the accuracy was 79.5% (74.1% to 84.3%). Analysis of the same radiographs by humans yielded a radiologists’ summary estimate sensitivity of 84.1% (81.0% to 87.0%), a specificity of 87.3% (85.0% to 89.3%), and an average accuracy of 84.8% (range 76.1-91.9%) (fig 2). The artificial intelligence candidate was ranked as the highest performing candidate in one mock examination (examination set 8) but came second to last overall across all interpretable images (rank 26/27).

Assuming the strictest marking criteria, which best reflects the actual FRCR examination (scenario 4), the overall sensitivity for the artificial intelligence candidate was 75.2% (67.4% to 81.9%), the specificity was 62.3% (54.0% to 70.0%), and the accuracy was 68.7% (63.1% to 73.9%). These compare with the radiologists’ summary estimates of sensitivity of 84.0% (80.8% to 86.7%), specificity of 87.5% (84.8% to 89.8%), and average accuracy of 85.2% (75.0-91.7%). In this scenario, the artificial intelligence candidate would rank last among its radiologist peers in half of all mock FRCR examinations and also last overall performance across all 300 radiographs across the 10 examinations is accounted for.

We allocated all certain and uncertain/doubtful positive findings by the artificial intelligence as the same. Of all the “certain” positive findings by the artificial intelligence, 92/104 (88%) were true positives; of the “uncertain” positive findings, 20/39 (51%) were true positives. If we excluded non-interpretable images, and considered only certain positive findings (with uncertain/doubtful positive findings assumed negative for pathology), the sensitivity would be reduced to 66.7% (60.1% to 76.4%), the specificity increased to 90.4% (83.8% to 94.4), and the accuracy rate similar at 79.2% (73.7% to 84.9%).

### Radiologist’ performance

Comparisons between the performance of the artificial intelligence candidate and the average radiologist’s performance across each mock examination are provided in table 2, figure 1, and figure 2. None of the 26 participating radiologists passed all 10 mock examinations. The highest performing radiologist passed 9/10 examinations (n=1); the lowest performing radiologists passed 1/10 examination (n=3). Ten (38%) radiologists passed at least five of the 10 mock examination sets. The average radiologist in this study was able to pass four of 10 mock examinations. Across all 300 radiographs included in the 10 mock examinations, the average radiologist’s mark was 255.6/300 (85%), with a median score of 257.5/300 (86%) and a range of 225-275 (75-92%).

### Radiologists’ subjective assessment of performance

Across all mock examinations, the radiologists rated each examination set as being slightly more difficult than the actual FRCR rapid reporting examination (average scores ranging between 6.0 and 7.4, where a score of 5 denotes a similar difficulty rating to the actual FRCR examination, a score of 10 denotes “far too difficult,” and a score of 1 denotes “far too easy”) **(** table 3). On average, radiologists scored their own performance across the mock examinations (out of 10) as being between 5.8 and 7.0 (not assuming that they had passed the mock examination, if the minimum pass mark is 9 out of 10) and scored the performance of the artificial intelligence candidate as being between 6.0 and 6.6 (assuming that the artificial intelligence candidate would score higher than themselves on average in three of 10 mock examinations).

**Table 3:** Summary of mean, median, range, and standard deviation of scores (Likert-type scale, 1-10) for radiologists’ opinions on difficulty level of each mock examination, their own performance, and that of AI tool

Examination	Mean score	Median score	Range	Standard deviation
**How representative of the FRCR was this examination?** *				
**1**	6.5	7.0	4-9	1.5
**2**	6.7	6.5	4-9	1.5
**3**	6.2	6.0	2-9	1.8
**4**	7.0	7.0	5-9	1.4
**5**	7.4	8.0	5-9	1.4
**6**	7.2	8.0	5-10	1.5
**7**	6.6	7.0	4-9	1.4
**8**	6.3	7.0	4-9	1.5
**9**	6.0	5.5	4-9	1.5
**10**	6.3	6.0	4-10	1.6
**How well do you think an AI model would perform on this examination?** **†**				
**1**	6.0	6.0	1-9	2.3
**2**	6.1	6.5	2-9	2.0
**3**	6.3	7.0	1-9	2.4
**4**	6.0	6.0	1-9	2.1
**5**	6.1	5.5	1-9	2.0
**6**	6.5	7.0	1-9	2.1
**7**	6.6	7.0	1-9	2.0
**8**	6.2	6.5	1-9	2.1
**9**	6.3	6.0	1-10	2.2
**10**	6.4	6.5	1-9	2.0
**How well do you think you performed on this examination?** **†**				
**1**	7.0	7.0	3-10	1.6
**2**	6.5	6.5	3-9	1.7
**3**	6.8	7.0	4-10	1.8
**4**	6.1	6.0	2-9	1.8
**5**	6.2	6.0	3-10	2.0
**6**	5.8	5.0	3-9	2.0
**7**	6.2	6.0	3-9	1.8
**8**	6.3	6.0	4-9	1.5
**9**	6.4	6.0	4-9	1.6
**10**	6.2	6.0	3-9	1.7
**Differences in scores between radiologists’ self-perception of performance and that of AI tool** **‡**				
**1**	1.0	1	−4-6	2.7
**2**	0.3	0	−4-6	2.5
**3**	0.5	0	−4-7	2.7
**4**	0.1	0	−5-6	2.3
**5**	0.0	0	−5-6	2.4
**6**	−0.7	0	−6-3	2.3
**7**	−0.5	0	−6-5	2.4
**8**	0.1	0	−4-5	2.3
**9**	0.1	0	−5-6	2.3
**10**	−0.2	0	−6-6	2.3

AI=artificial intelligence; FRCR=Fellowship of the Royal College of Radiologists.

* 1=too easy; 5=about right; 10=too difficult.

† 1=everything incorrect; 5=half correct; 10=perfect, everything correct.

‡ Negative scores denote perception of AI performing better.

### Imaging pitfalls

**Fig 3:**
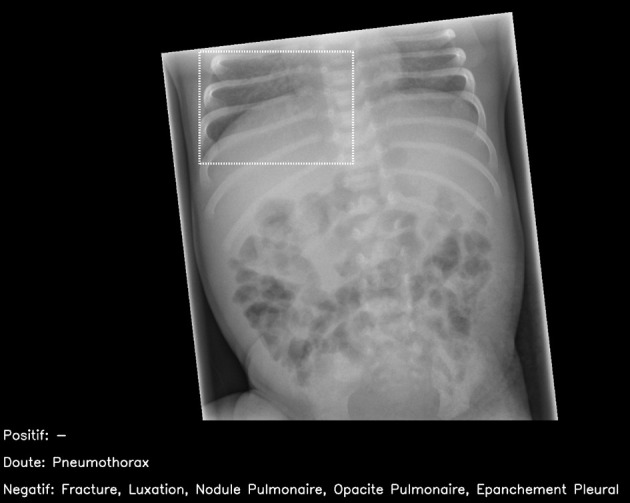
Normal paediatric abdominal radiograph interpreted by artificial intelligence (AI) candidate as having right basal pneumothorax with dashed bounding box (false positive result). This should have been identified as non-interpretable by AI. French translation: positif=positive; doute=doubt; epanchement pleural=pleural effusion; luxation=dislocation; negatif=negative; nodule pulmonaire=pulmonary nodule; opacite pulmonaire=pulmonary opacification

**Fig 4:**
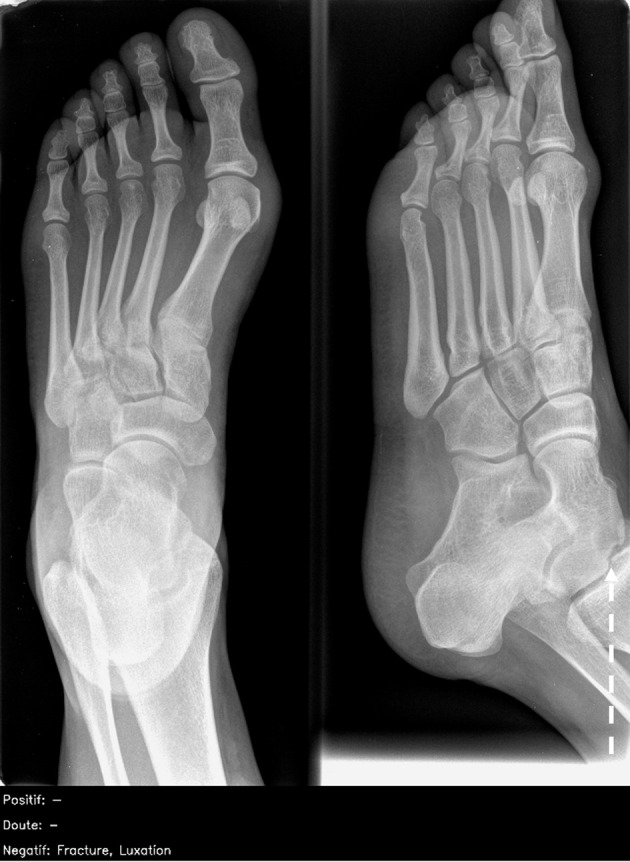
Dorsoplantar and oblique views of abnormal right foot radiograph in adult showing osteochondral defect at talar dome (white dashed arrow). This finding was missed by all 26 radiologists and also artificial intelligence candidate (false negative) and was particularly challenging. French translation: doute=doubt; luxation=dislocation; negatif=negative; positif=positive

**Fig 5:**
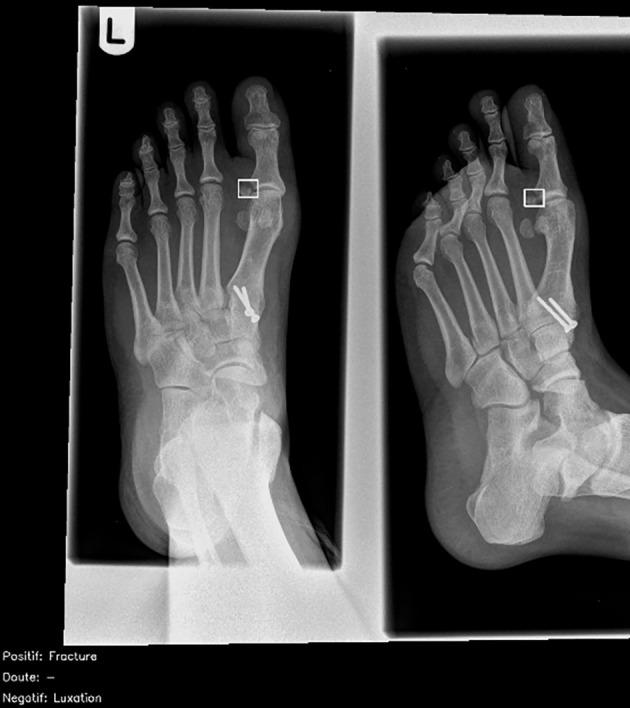
Dorsoplantar and oblique views of abnormal right foot radiograph in adult showing acute fracture of proximal phalanx of big toe, correctly interpreted by less than half of radiologists (46%) and correctly identified by artificial intelligence candidate (dashed bounding box). French translation: doute=doubt; luxation=dislocation; negatif=negative; positif=positive

**Fig 6:**
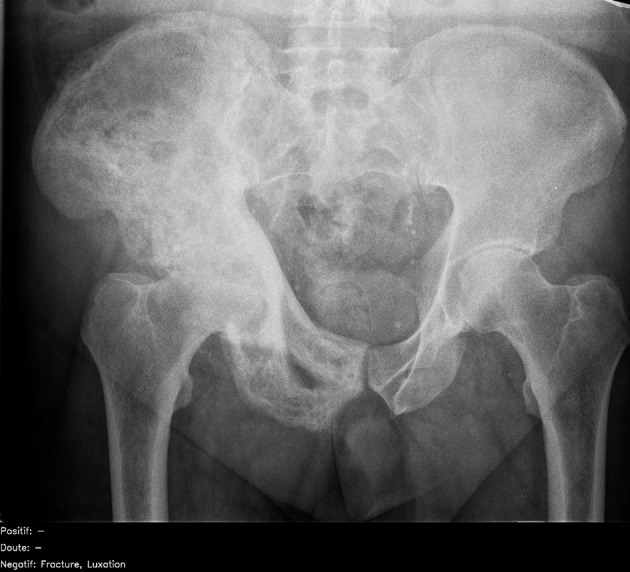
Abnormal adult pelvic radiograph showing increased sclerosis and expansion of right iliac bone in keeping with Paget’s disease. This was correctly identified by almost all radiologists (96%) but interpreted as normal by artificial intelligence candidate (false negative), given that this was not a pathology it was trained to identify. French translation: doute=doubt; luxation=dislocation; negatif=negative; positif=positive

**Fig 7:**
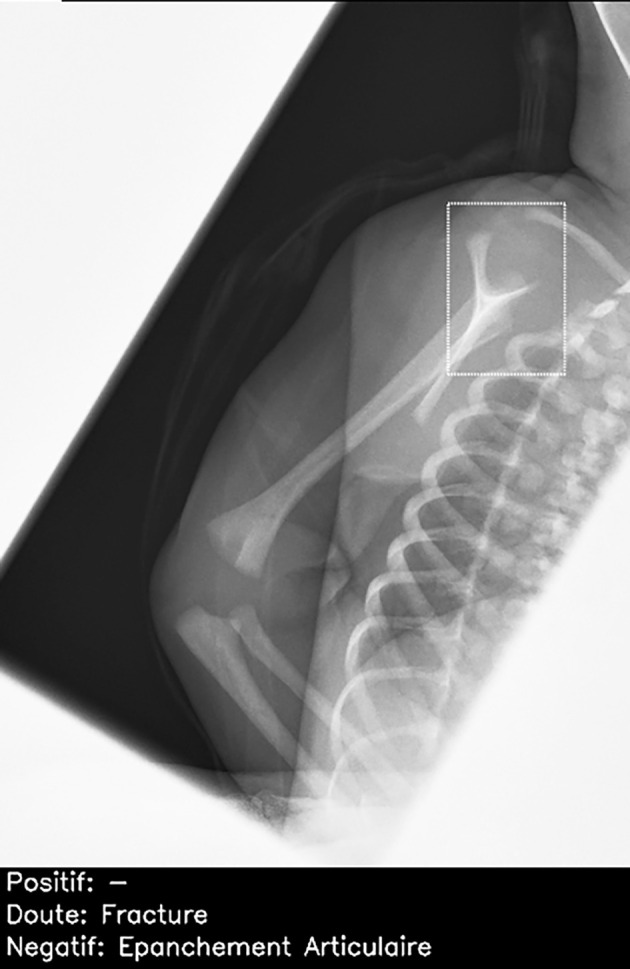
Normal lateral scapular Y view of right shoulder in child, incorrectly interpreted by artificial intelligence candidate as having proximal humeral fracture (dashed bounding box). This was false positive result, which was correctly identified as normal by all 26 radiologists. French translation: doute=doubt; epanchement articulaire=joint effusion; negatif=negative; positif=positive

Table 4 provides a summary of the imaging pitfalls, and figures 3-7 show a selection of imaging examples.

**Table 4:** Summary of imaging pitfalls by artificial intelligence (AI) tool compared with human performance

AI interpretation	No	Body part	Diagnosis	No (%) radiologists correct
**Non-interpretable images (n=42)**				
False positive (uncertain)	1	Abdomen	Basal pneumothorax	21 (81)
**Images most radiologists (>50%) diagnosed incorrectly (n=20)**				
False positive (uncertain)	1	Hand	Index finger bone lesion	8 (31)
False negative	9	Knee	Lateral tibial spine fracture	11 (42)
		Pelvis	Left iliac wing fracture	10 (38)
		Chest	Pulmonary nodule	8 (31)
		Elbow	Coracoid fracture	8 (31)
		Foot	Second metatarsal fracture	8 (31)
		Scaphoid	Fourth metacarpal fracture	5 (19)
		Clavicle	Medial clavicular fracture	3 (12)
		Ankle	Distal fibular fracture	3 (12)
		Foot	Talar osteochondral defect	0
True positive (certain)	5	Foot	Fracture proximal phalanx of big toe	12 (46)
		Chest	Apical pneumothorax	11 (42)
		Chest	Multiple rib fractures	9 (35)
		Foot	Fracture distal phalanx of big toe	7 (27)
		Scaphoid	Scaphoid fracture	0
True positive (uncertain)	3	Foot	Navicular fracture	11 (42)
		Shoulder	Anterior shoulder dislocation	10 (38)
		Hand	Hamate fracture	7 (27)
True negative	2	Pelvis	None	12 (46)
		Foot	None	5 (19)
**Images almost all radiologists (>90%) diagnosed correctly (n=148)**				
False positive (certain)	4	Foot	Fifth metatarsal fracture	25 (96)
		Knee	Fractured patella	24 (92)
		Chest	Right lower lobe consolidation	24 (92)
		Shoulder	Glenohumeral dislocation	24 (92)
False positive (uncertain)	6	Chest	Parenchymal opacity	26 (100)
		Chest	Right lower lobe consolidation	26 (100)
		Humerus	Proximal humeral fracture	26 (100)
		Chest	Right lower lobe consolidation	25 (96)
		Foot	Cuboidal fracture	25 (96)
		Hand	Proximal phalangeal fracture of thumb	25 (96)
False negative	4	Foot	Second metatarsal fracture	26 (100)
		Foot	Second metatarsal fracture	26 (100)
		Hip	Paget’s disease	25 (96)
		Pelvis	Avulsion fracture	25 (96)
True positive (certain)	58	For brevity, detailed review of these 134 correctly diagnosed radiographs by AI candidate is not provided		
True positive (uncertain)	9			
True negatives	67			

#### Non-interpretable images

Forty two (14%) radiographs in the dataset should be non-interpretable by the commercial artificial intelligence tool. Of these, one (2%) yielded a result by the artificial intelligence. This was mislabelled as a basal pneumothorax on a normal paediatric abdominal radiograph (fig 3).

#### Images that most radiologists diagnosed wrongly

Twenty (7%) radiographs were incorrectly diagnosed by more than half of the radiologists. Of these, half (10/20; 50%) were also incorrectly diagnosed by the artificial intelligence (nine false negatives (fig 4); one false positive), which were mostly missed musculoskeletal findings (8/10; 80%). The remaining 10/20 (50%) radiographs were correctly diagnosed by the artificial intelligence (fig 5), of which 6/10 (50%) were related to radiographs of the extremities (hands, carpal bones, feet).

#### Images diagnosed correctly by almost all radiologists

One hundred and forty eight (49%) radiographs were correctly diagnosed by >90% of the radiologists. Of these, 134/148 (91%) were also correctly diagnosed by the artificial intelligence candidate (67 true negatives; 67 true positives). The artificial intelligence candidate was incorrect for 14/148 (9%) radiographs. These included 4/14 (29%) false negative diagnoses (fig 6) and 10/14 (71%) false positive diagnoses (fig 7). In 10/14 (71%) cases, the errors were made on musculoskeletal radiographs.

## Discussion

To our knowledge, this is the first time an “artificial intelligence candidate” has been invited to take part in a (mock) FRCR examination. Assuming that the college would provide special dispensation to take account of the artificial intelligence candidate’s lack of experience in interpreting skull, spine, and abdominal radiographs, a pass mark (>90% correct) would have been achieved in two of the 10 mock examinations. However, if dispensation was not granted, the artificial intelligence candidate would not pass any of the mock examinations. In this scenario, the artificial intelligence candidate would be best served by adopting a strategy of assigning non-interpretable images as “normal,” which would have allowed it to pass one of the 10 mock examinations. The artificial intelligence candidate can take solace in the fact that this was a particularly challenging set of rapid reporting examinations, with the average human radiologist counterpart passing only four of the 10 mock examinations. If these examination marks were moderated to account for the level of difficulty, as they would be in the real FRCR examination, the pass rates may have been higher.

Although the artificial intelligence candidate did not outperform most of the radiologists, its accuracy was relatively high considering the case mix and complexity. For the radiographs in which image analysis was possible, the artificial intelligence candidate had a sensitivity of 83.6%, a specificity of 75.2%, and an overall accuracy of 79.5% (compared with the summary estimate of radiologists with a sensitivity of 84.1%, a specificity of 87.3%, and an average accuracy of 85.2%). The artificial intelligence candidate was ranked as the highest scoring candidate for one of the mock examinations and outperformed three radiologists who passed only one mock examination (the artificial intelligence candidate passed two). Nevertheless, the artificial intelligence candidate would still need further training to achieve the same level of performance and skill of an average recently FRCR qualified radiologist, particularly in the identification of subtle musculoskeletal abnormalities (which made up the majority of the artificial intelligence imaging pitfalls) and also in interpretation of abdominal, skull, and spine radiographs, which it has no training or experience in analysing. The artificial intelligence candidate was, however, correct in its diagnosis in half of the cases that most radiologists failed, particularly when these involved hands and feet. These radiographs probably contain more bones and joints for evaluation, which humans may find time consuming and tedious but an artificial intelligence would not.

### Comparison with other studies

The artificial intelligence candidate’s performance is representative of similar artificial intelligence models reported in the wider literature. A recent meta-analysis of artificial intelligence algorithms for detecting fractures on imaging reported a sensitivity of 89% and a specificity of 80% in studies with adequate external validation cohorts and low risk of bias.^11^ Another meta-analysis of artificial intelligence algorithms for classifying abnormal versus normal chest radiographs found a sensitivity and specificity of 87% and 89%, respectively^12^; however, this also included studies without external validation, which will likely boost the accuracy reported in the meta-analysis. These studies also did not require a diagnosis to be provided, just a binary distinction of normality versus abnormality, unlike our study. Radiographs incorporated in the mock examinations in our study were chosen to challenge radiologists at the peri-consultant level, whereas datasets typically used to train and test artificial intelligence algorithms include the full range of clinical findings from the very easy to the very hard, inevitably boosting their overall performance.

### Strengths of study

This study provides one of the more comprehensive cross comparisons between radiologists and artificial intelligence, including 26 readers and 300 radiographs from an external dataset not previously seen by the artificial intelligence candidate. The closest comparator is a study examining the use of artificial intelligence in augmenting the accuracy of reporting of chest radiographs, which looked at 20 readers and 4568 radiographs but examined performance in an internal testing cohort rather than looking at external validation. This point is important as diagnostic performance is known to drop in external datasets; a recent systematic review showed that 80% of algorithms’ performance dropped in external test sets, with 24% having a substantial (>10%) reduction.^13^

The case load difficulty is both a strength and a weakness of our analysis. Despite all readers having recently passed the FRCR examination, each reader passed only four of 10 examinations (pass mark set as 90%), ranging from one to nine of 10 examinations passed across the 26 radiologist readers. Thus the cases we used are almost certainly more difficult than those provided in the actual FRCR examination. This is also evidenced by subjective feedback from the radiologist participants. Although it slightly reduces the generalisation of the results to the true FRCR examinations, this augments rather than detracts from the comparison in performance, as it focuses on the challenging cases encountered in clinical practice by reporting radiologists. Nevertheless, we must remember that in real world settings, many software as medical device algorithms can be “unlocked” after approval by the US Food and Drug Administration to permit additional model training in real world settings to correct for biases and help to improve local performance where needed. Although the artificial intelligence performed well in the test sets for cases it could interpret, it performed poorly compared with the human readers when being marked according to same criteria as radiologists for the FRCR examination. It was ranked as the bottom performing candidate in half of the mock examinations and also last overall; however, one must remember that the artificial intelligence candidate was never trained or intended to complete any radiology mock examinations alone, let alone act autonomously in clinical practice.

### Policy and clinical implications

The promise of artificial intelligence as a diagnostic adjunct in clinical practice remains high. Although lowly ranked for diagnostic accuracy rate overall (rank 26), the artificial intelligence came close to radiologist level performance when we consider the cases it could interpret. This could potentially bring near radiologist level accuracy to physicians in the clinical environment (especially considering that the radiologists in this cohort could potentially be higher performing, given recent examination success) and where routine immediate radiographic reporting by non-radiologists is not available and levels of training in and exposure to radiographic interpretation can be highly heterogeneous. Previous work on analysis of chest radiographs has also shown a beneficial effect of the addition of artificial intelligence assistance on the accuracy of reporting radiologists, with the area under the curve improving from 0.71 for radiologists reporting on their own to 0.81 for those reporting in conjunction with an artificial intelligence tool, as well as reducing the reading time needed to generate a report.^14^ In another study, artificial intelligence was also shown to improve radiologists’ sensitivity by 10.4% (versus without artificial intelligence) for interpreting musculoskeletal radiographs and to reduce reporting time by 6.3 seconds per examination.^15^ Nevertheless, the artificial intelligence should analyse only radiographs that it has been trained to interpret to avoid erroneous anomalies being highlighted on those deemed non-interpretable, which occurred in one case in this study (and has been known to occur with other artificial intelligence tools).^16^

Interestingly, in this study, radiologists slightly overestimated the likely performance of the artificial intelligence candidate, assuming that it would perform almost as well as themselves on average and outperform them in at least three of the 10 mock examinations. This was not the case. Although this reputation is flattering for the artificial intelligence candidate, it may be dangerous in situations where radiologists call upon and rely on the artificial intelligence for performing their daily tasks without supervision (known in one study to occur in 18% of cases in which artificial intelligence was used for assessment of bone age on radiographs,^17^ despite not being intended for autonomous usage). Alternatively, radiologists (and other healthcare professionals) may become overly influenced by the artificial intelligence, prioritising its opinion over their own owing to this underlying cognitive bias. Further education, awareness, and transparency about imaging pitfalls (sometimes referred to as “failure analysis”) should be highlighted by the artificial intelligence candidate to its radiologist peers, so that unreasonable expectations are not placed on its workload and patients’ safety remains paramount.^18^ ^19^ In a future scenario, in which the performance of artificial intelligence reaches that of humans and artificial intelligence is widely adopted in clinical practice for radiographic interpretation, radiologists’ training may place a greater focus on the evaluation of radiographs for which artificial intelligence yields inaccurate or uninterpretable results (the college could potentially use an artificial intelligence to select out such cases for rapid reporting examinations). Alternatively, the rapid reporting examination component may be abolished entirely for more complex reasoning tasks (for example, resolution of differential diagnoses based on challenging imaging) should radiographic interpretation no longer play a large role in the radiologists’ job.

### Limitations of study

Our study has several limitations. Although we aimed to determine whether an artificial intelligence tool (“candidate”) could pass the FRCR rapid reporting examination, we were unable to acquire actual past examinations and therefore had to use mock examinations as a proxy. As discussed above, these were likely more difficult than those provided in the actual examination. In addition, in the FRCR examination, results are closely moderated to ensure that they are representative of all current and previous examinations, and precise pass marks may be modified if a particular examination test set is discovered to be overly challenging. We elected to provide the raw data as we received them here for review rather than try to adjust the pass marks.

A second limitation is the lack of overall control and supervision of the radiologists by the study investigators during their interpretation of the images. Clear instructions were set regarding interpretation, with images provided via an online DICOM viewer, but radiologists were not directly timed or supervised. Some may have been distracted during their imaging interpretation, may not have done the interpretation under ideal conditions using a suitable monitor with dim lighting, or may not have felt as much pressure to do their best as one would in a real examination. This could explain some of the lower than expected scores returned by the radiologists. Nevertheless, the high number of radiologist readers included in this study provided a broad range of scores and results for analysis. Finally, we have examined only the FRCR examination structure, so we cannot comment on generalisation to other examination boards or on the likely success of artificial intelligence in these. Finally, we evaluated only one artificial intelligence tool in this study, which we believe to be representative of the tools available on the basis of results of published meta-analyses. Further work on how well other commercial and non-commercial artificial intelligence tools (used either as standalone products or in combination) would be an interesting avenue for further work.

### Conclusion

On this occasion, the artificial intelligence candidate was unable to pass any of the 10 mock examinations when marked against similarly strict criteria to its human counterparts, but it could pass two of the mock examinations if special dispensation was made by the RCR to exclude images that it had not been trained on. Further training and revision are strongly recommended, particularly for cases the artificial intelligence considers “non-interpretable,” such as abdominal radiographs and those of the axial skeleton. Increased familiarity with less common and more subtle bony pathologies will also help to boost the chances of examination success.

## What is already known on this topic

A large number approved commercial artificial intelligence (AI) products are available on the market, many of which are suited for “narrow” (ie, specific) AI tasks
For acute chest and musculoskeletal radiographic interpretation, the diagnostic accuracy of AI has been shown to be high, with sensitivity rates of 86% and 89%, respectively
Most diagnostic accuracy tests assessing AI performance have been applied to simpler diagnoses than radiologists would be expected to interpret in their final rapid reporting examination

## What this study adds

The AI candidate was able to pass two of the 10 mock examinations, compared with an average of four mock examinations by trainees who had recently passed the examination
Overall sensitivity, specificity, and accuracy rates were high for images that the AI could interpret at 83.6%, 75.2%, and 79.5% respectively
Summary estimates across 26 radiologist readers were 84.1%, 87.3%, and 84.8%, respectively

## Data Availability

The data are not publicly available. Requests for the anonymised imaging dataset will be considered and should be sent to the corresponding author at susan.shelmerdine@gosh.nhs.uk.

## Web extra

Extra material supplied by authors

Web appendix: Supplementary materials

## Notes

FRCR-AI Study Collaborators: Sayed Hashim AlQarooni, Sajay Alias, Saraswati Samyukta Aryasomayajula, Caline Azzi, Awadh Ahmed Taiseir Ba Hashwan, Anintitha Boon-Itt, Momana Tariq Butt, Liisa Chang, Victoria Crowe, Mahmoud Mohamed Elsaidy, Aejaz Ahmed Gonegandla, Abeeku Afedzi Hammond, Mansi Jantre, Kavya Moonjelil Karthikeyan, Sharon Koo, Sophie McGlade, Aprajita Mehta, Preeti Mundhada, Marwa Nayel, Wee Ping Ngu, Ryan Norman, Amina Odeh, Dileep Kumar Perumala, Shayeri Roy Choudhury, Sarath Babu Stephen Maria Lourdam, Anil Kumar Geetha Virupakshappa.

The lead authors (the manuscript’s guarantors) affirm that the manuscript is honest, accurate, and transparent account of the study being reported; that no important aspects of the study have been omitted; and that any discrepancies from the study as planned (and, if relevant, registered) have been explained.

Dissemination to participants and related patient and public communities: Results were sent to all radiologist readers after they took part. The results will be shared with the wider radiology and medical community through social media channels, educational meetings, and discussion with the educational committee of the Royal College of Radiologists, as well as the PPI groups.

Provenance and peer review Not commissioned; externally peer reviewed.
