# Supplementary material for: Can artificial intelligence pass the Fellowship of the Royal College of Radiologists examination? Multi-reader diagnostic accuracy study
Source: BMJ. 2022 Dec 21;379:e072826. doi: 10.1136/bmj-2022-072826 (PMC9768816; doi:10.1136/bmj-2022-072826)
Supplement: Supplementary file 1 — Web appendix: Supplementary materials [file shes072826.ww1.pdf]

Supplementary Material

Supplementary Table A

The spread of normal and abnormal cases (divided into those of adult and paediatric radiographs) across the ten mock rapid reporting examination sets provided to the AI model and radiologists.

Although radiologists are aware that approximately half of the cases per mock examination are abnormal (as per the real FRCR examination), the exact number of these are unknown to them when sitting the examination. There is no predefined reported split of paediatric versus adult cases in the examination. The denominator for percentages calculated in brackets are derived from 'total radiographs' in the final column.

Mock Set	Paediatric Cases (%)	Adult Cases	Total Normal (%)	Total Abnormal (%)	Total Radiographs
1	3 (10.0)	27 (90.0)	14 (46.7)	16 (53.3)	30
2	9 (30.0)	21 (70.0)	15 (50.0)	15 (50.0)	30
3	4 (13.3)	26 (86.7)	12 (40.0)	18 (60.0)	30
4	7 (23.3)	23 (76.7)	12 (40.0)	18 (60.0)	30
5	3 (10.0)	27 (90.0)	12 (40.0)	18 (60.0)	30
6	5 (16.7)	25 (83.3)	17 (56.7)	13 (43.3)	30
7	3 (10.0)	27 (90.0)	16 (53.3)	14 (46.7)	30
8	11 (36.7)	19 (63.3)	16 (53.3)	14 (46.7)	30
9	6 (20.0)	24 (80.0)	15 (50.0)	15 (50.0)	30
10	7 (23.3)	23 (76.7)	13 (43.3)	17 (56.7)	30
**Total across 10 mock examinations**	**58 (19.3)**	**242 (80.7)**	**142 (47.3)**	**158 (52.7)**	**300**

Supplementary Table B

Distribution of normal and abnormal radiographs according to body parts (listed alphabetically) across the 10 mock FRCR examinations, split into those that were adult and paediatric cases. This table demonstrates the range of type of cases included in this study.

Body Part	Normal Paediatric	Normal Adult	Abnormal Paediatric	Abnormal Adult	Total Paediatric	Total Adult	Total Normal	Total Abnormal	Total Overall per body part
Abdomen	3	7	1	2	4	9	10	3	13
Ankle	1	7	1	4	2	11	8	5	13
Calcaneus	0	1	0	0	0	1	1	0	1
Chest	3	21	2	24	5	45	24	26	50
Clavicle	0	1	0	2	0	3	1	2	3
Cervical Spine	1	3	0	3	1	6	4	3	7
Elbow	2	4	2	6	4	10	6	8	14
Facial Bones	0	4	0	3	0	7	4	3	7
Femur	0	1	0	0	0	1	1	0	1
Fingers	0	5	0	2	0	7	5	2	7
Foot	3	7	1	23	4	30	10	24	34
Hand	5	1	3	14	8	15	6	17	23
Hip	2	1	1	2	3	3	3	3	6
Humerus	1	0	0	0	1	0	1	0	1
Knee	1	8	0	6	1	14	9	6	15
Lumbar Spine	0	4	0	1	0	5	4	1	5
Mandible	1	1	0	0	1	1	2	0	2
Neck Soft Tissues	0	0	0	3	0	3	0	3	3
Orthopantogram	1	0	0	1	1	1	1	1	2
Paranasal Sinsuses	0	0	0	1	0	1	0	1	1
Pelvis	3	5	2	6	5	11	8	8	16
Radius/Ulna	3	0	2	0	5	0	3	2	5
Sacro-iliacs	0	1	0	0	0	1	1	0	1
Scaphoid Views	0	2	0	8	0	10	2	8	10
Scapula	0	1	0	0	0	1	1	0	1
Shoulder	1	9	1	13	2	22	10	14	24
Thoracic Spine	0	2	0	0	0	2	2	0	2
Thumb	0	4	0	1	0	5	4	1	5
Tibia/Fibula	2	0	3	1	5	1	2	4	6

Toes	0	1	0	4	0	5	1	4	5
Wrist	2	6	4	5	6	11	8	9	17
**Grand Total**	**35**	**107**	**23**	**135**	**58**	**242**	**142**	**158**	**300**

Supplementary Table C

Confusion matrix demonstrating performance of the commercial AI model per body part across all 300 radiographs included in this study. Due to the small number of cases per body part, diagnostic accuracy rates were not calculated but numbers provided here are for full granularity of data. *NI – non-interpretable, TP – true positive, FP – false positive, TN – true negative, FN – false negative.*

Body Part	NI	TP		FP		TN	FN
		Certain	Uncertain	Certain	Uncertain		
Abdomen	12	0	0	0	1	0	0
Ankle	0	3	0	1	0	7	2
Calcaneus	0	0	0	0	0	1	0
Chest	0	18	3	2	5	20	2
Clavicle	0	1	0	0	0	1	1
Cervical Spine	7	0	0	0	0	0	0
Elbow	0	7	0	0	0	6	1
Facial Bones	7	0	0	0	0	0	0
Femur	0	0	0	0	1	0	0
Fingers	0	1	1	0	0	5	0
Foot	0	12	3	1	2	8	8
Hand	0	11	4	0	2	5	1
Hip	0	1	1	0	0	3	1
Humerus	0	0	0	0	1	0	0
Knee	0	3	1	2	1	7	1
Lumbar Spine	5	0	0	0	0	0	0
Mandible	2	0	0	0	0	0	0
Neck Soft Tissues	3	0	0	0	0	0	0
Orthopantogram	2	0	0	0	0	0	0
Paranasal Sinsuses	1	0	0	0	0	0	0
Pelvis	0	4	2	0	0	8	2
Radius/Ulna	0	1	1	0	1	2	0
Sacro-iliacs	0	0	0	0	0	1	0
Scaphoid Views	0	7	0	1	1	0	1
Scapula	0	0	0	0	0	1	0
Shoulder	0	8	3	2	3	6	2

Thoracic Spine	2	0	0	0	0	0	0
Thumb	0	0	0	1	1	3	0
Tibia/Fibula	0	3	1	0	0	2	0
Toes	0	3	0	1	0	1	0
Wrist	0	9	0	1	0	7	0
**Grand Total**	**41**	**92**	**20**	**12**	**19**	**94**	**22**

Supplementary Table D

Examination scores and diagnostic accuracy rates for each mock examination (and also overall performance) for the commercially available AI model, divided across the three different methods of examination marking.

For calculation of examination diagnostic accuracy rates in this study, for scenario 1 all non-interpretable radiographs were excluded from analysis, in scenario 2 all non-interpretable radiographs were assumed to be normal (i.e. normal = TN, abnormal = FN), in scenario 3 all non-interpretable radiographs were assumed to be abnormal (i.e. normal = FP, abnormal = TP) and scenario 4 all non-interpretable radiographs were considered wrongly interpreted (i.e. normal = FN, abnormal = FP).

Pass and fail outcomes are assigned according to pass mark of 90% or above.

**SCENARIO 1**							
**Exam Set**	**Exam Score**	**Sensitivity (95% CI)**	**Specificity (95% CI)**	**PPV (95% CI)**	**NPV (95% CI)**	**Accuracy (95% CI)**	**Pass/Fail**
**1**	22 / 27	76.9 (46.2, 94.9)	85.7 (57.2, 98.2)	83.3 (57.3, 94.9)	80.0 (59.2, 91.7)	81.5 (61.9, 93.7)	FAIL
**2**	16 / 23	72.7 (39.0, 93.9)	20.0 (0.5, 71.6)	66.7 (53.1, 77.9)	25.0 (4.3, 71.2)	56.3 (29.9, 80.3)	FAIL
**3**	19 / 25	93.3 (68.1, 99.8)	50.0 (18.7, 813)	73.7 (59.8, 84.1)	83.3 (40.5, 97.4)	76.0 (54.9, 90.6)	FAIL
**4**	18 / 24	80.0 (51.9, 95.7)	66.7 (29.9, 92.5)	80.0 (60.6, 91.3)	66.7 (39.7, 85.9)	75.0 (53.3, 90.2)	FAIL
**5**	15 / 26	73.3 (44.9, 92.2)	36.4 (10.9, 69.2)	61.1 (47.8, 72.9)	50.0 (24.1, 75.9)	57.7 (36.9, 76.7)	FAIL
**6**	22 / 27	84.6 (54.6, 98.1)	78.6 (49.2, 95.3)	78.6 (56.7, 91.1)	84.6 (59.9, 95.3)	81.5 (61.9, 93.7)	FAIL
**7**	22 / 26	83.3 (51.6, 97.9)	85.7 (57.2, 98.2)	83.3 (57.5, 94.9)	85.7 (62.5, 95.6)	84.6 (65.1, 95.6)	FAIL
**8**	26 / 27	100.0 (71.5, 100.0)	93.4 (69.8, 99.8)	91.7 (62.3, 98.7)	100.0 (-)	96.3 (81.0, 99.9)	**PASS**
**9**	23 / 29	78.6 (49.2, 95.3)	80.0 (51.9, 95.7)	78.6 (56.2, 91.3)	80.0 (58.7, 91.8)	79.3 (60.3, 92.0)	FAIL
**10**	23 / 25	93.3 (68.1, 99.8)	90.0 (55.5, 99.8)	93.3 (68.5, 98.9)	90.0 (57.3, 98.4)	92.0 (73.9, 99.0)	**PASS**
**Total**	206 / 259	83.6 (76.2, 89.4)	75.2 (66.7, 82.5)	78.3 (72.5, 83.2)	81.0 (74.2, 86.4)	79.5 (74.1, 84.3)	FAIL

SCENARIO 2							
**Exam Set**	**Exam Score**	**Sensitivity (95% CI)**	**Specificity (95% CI)**	**PPV (95% CI)**	**NPV (95% CI)**	**Accuracy (95% CI)**	**Pass/Fail**
**1**	24 / 30	71.4 (41.9, 91.6)	87.5 (61.7, 98.5)	83.3 (56.7, 95.0)	77.8 (60.0, 89.1)	80.0 (61.4, 92.3)	FAIL
**2**	20 / 30	57.1 (28.9, 82.3)	75.0 (47.6, 92.7)	66.7 (43.3, 84.0)	66.7 (50.6, 79.6)	66.7 (47.2, 82.7)	FAIL
**3**	22 / 30	82.4 (56.6, 96.2)	82.4 (56.6, 96.2)	73.7 (57.6, 85.2)	72.7 (46.7, 89.0)	73.3 (54.1, 87.7)	FAIL
**4**	22 / 30	70.6 (44.0, 89.7)	76.9 (46.2, 95.0)	80.0 (58.6, 91.9)	66.7 (47.5, 81.6)	73.3 (54.1, 87.7)	FAIL
**5**	18 / 30	68.8 (41.3, 88.9)	50.0 (23.0, 77.0)	61.1 (45.8, 74.5)	58.3 (36.4, 77.4)	60.0 (40.6, 77.3)	FAIL
**6**	25 / 30	84.6 (54.6, 98.1)	82.4 (56.6, 96.2)	78.6 (56.1, 91.3)	87.5 (65.8, 96.2)	83.3 (65.3, 94.4)	FAIL
**7**	24 / 30	71.4 (41.9, 91.6)	87.5 (61.7, 98.5)	83.3 (56.7, 95.0)	77.8 (60.0, 89.1)	80.0 (61.4, 92.3)	FAIL
**8**	26 / 30	78.6 (49.2, 95.3)	93.8 (69.8, 99.8)	91.7 (61.8, 98.7)	83.3 (64.5, 93.2)	86.7 (69.3, 96.2)	FAIL
**9**	24 / 30	78.6 (49.2, 95.3)	81.3 (54.5, 96.0)	78.6 (56.1, 91.3)	81.3 (60.7, 92.4)	80.0 (61.4, 92.3)	FAIL
**10**	27 / 30	87.5 (61.7, 98.5)	92.9 (66.1, 99.8)	93.3 (67.7, 98.9)	86.7 (63.8, 96.0)	90.0 (73.5, 97.9)	**PASS**
**Total**	232 / 300	75.2 (67.4, 81.9)	79.5 (72.1, 85.6)	78.3 (72.3, 83.4)	76.4 (70.8, 81.3)	77.3 (72.2, 81.9)	FAIL
**SCENARIO 3**							
**Exam Set**	**Exam Score**	**Sensitivity (95% CI)**	**Specificity (95% CI)**	**PPV (95% CI)**	**NPV (95% CI)**	**Accuracy (95% CI)**	**Pass/Fail**
**1**	23 / 30	78.6 (49.2, 95.3)	75.0 (47.6, 92.7)	73.3 (53.0, 87.0)	80.0 (58.5, 91.9)	76.7 (57.7, 90.1)	FAIL
**2**	19 / 30	78.6 (49.2, 95.3)	50.0 (24.7, 75.4)	57.9 (44.0, 70.7)	72.7 (46.6, 89.1)	63.3 (43.9, 80.1)	FAIL
**3**	21 / 30	94.1 (71.3, 99.9)	38.5 (13.9, 68.4)	66.7 (56.2, 75.8)	83.3 (39.8, 97.4)	70.0 (50.6, 85.3)	FAIL
**4**	20 / 30	82.4 (56.6, 96.2)	46.2 (19.2, 74.9)	66.7 (53.6, 77.6)	66.7 (38.0, 86.7)	66.7 (47.2, 82.7)	FAIL
**5**	16 / 30	75.0	28.6	54.6	50.0	53.3	FAIL

		(47.6, 92.7)	(8.4, 58.1)	(43.7, 65.0)	(23.4, 76.6)	(34.3, 71.7)	
**6**	22 / 30	84.6 (54.6, 98.1)	64.7 (38.3, 85.8)	64.7 (48.1, 78.4)	84.6 (59.5, 95.4)	73.3 (54.1, 87.7)	FAIL
**7**	24 / 30	85.7 (57.2, 98.2)	75.0 (47.6, 92.7)	75.0 (55.6, 87.8)	85.7 (61.7, 95.7)	80.0 (61.4, 92.3)	FAIL
**8**	29 / 30	100.0 (76.8, 100.0)	93.8 (69.8, 99.8)	93.3 (67.7, 98.9)	100.0 (-)	96.7 (82.8, 99.9)	FAIL
**9**	23 / 30	78.6 (49.2, 95.3)	75.0 (47.6, 92.7)	73.3 (53.0, 87.0)	80.0 (58.5, 91.9)	76.7 (57.7, 90.1)	FAIL
**10**	24 / 30	93.8 (69.8, 99.8)	64.3 (35.1, 87.2)	75.0 (59.5, 86.0)	90.0 (56.5, 98.4)	80.0 (61.4, 92.3)	FAIL
**Total**	221 / 300	85.2 (78.5, 90.5)	62.3 (54.0, 70.0)	69.0 (64.2, 73.4)	81.0 (74.0, 86.5)	73.7 (68.3, 78.6)	FAIL
**SCENARIO 4**							
**Exam Set**	**Exam Score**	**Sensitivity (95% CI)**	**Specificity (95% CI)**	**PPV (95% CI)**	**NPV (95% CI)**	**Accuracy (95% CI)**	**Pass/Fail**
**1**	22 / 30	71.4 (41.9, 91.6)	75.0 (47.6, 92.7)	71.4 (50.1, 86.1)	75.0 (55.6, 87.8)	73.3 (54.1, 87.7)	FAIL
**2**	16 / 30	57.1 (28.9, 82.3)	50.0 (24.7, 75.4)	50.0 (33.9, 66.1)	57.1 (37.9, 74.4)	53.3 (34.3, 71.7)	FAIL
**3**	19 / 30	82.4 (56.6, 96.2)	38.5 (13.9, 68.4)	63.6 (51.9, 73.9)	62.5 (32.6, 85.2)	63.3 (43.9, 80.1)	FAIL
**4**	18 / 30	70.6 (44.0, 89.7)	46.2 (19.2, 74.9)	63.2 (48.7, 75.6)	54.6 (31.9, 75.5)	60.0 (40.6, 77.3)	FAIL
**5**	15 / 30	68.8 (41.3, 89.0)	28.6 (8.4, 58.1)	52.4 (40.8, 63.7)	44.4 (21.0, 70.7)	50.0 (31.3, 68.7)	FAIL
**6**	22 / 30	84.6 (54.6, 98.1)	64.7 (38.3, 85.8)	64.7 (48.1, 78.4)	84.6 (59.5, 95.4)	73.3 (54.1, 87.7)	FAIL
**7**	22 / 30	71.4 (41.9, 91.6)	75.0 (47.6, 92.7)	71.4 (50.1, 86.1)	75.0 (55.6, 87.8)	73.3 (54.1, 87.7)	FAIL
**8**	26 / 30	78.6 (49.2, 95.3)	93.8 (69.8, 99.8)	91.7 (61.8, 98.7)	83.3 (64.5, 93.2)	86.7 (69.3, 96.2)	FAIL
**9**	23 / 30	78.6 (49.2, 95.3)	75.0 (47.6, 92.7)	73.3 (53.0, 87.0)	80.0 (58.5, 91.9)	76.7 (57.7, 90.1)	FAIL

**10**	23 / 30	87.5 (61.7, 98.5)	64.3 (35.1, 87.2)	73.7 (57.5, 85.3)	81.8 (53.8, 94.6)	76.7 (57.7, 90.1)	FAIL
**Total**	206 / 300	75.2 (67.4, 81.9)	62.3 (54.0, 70.0)	66.3 (61.1, 71.1)	71.8 (65.2, 77.5)	68.7 (63.1, 73.9)	FAIL

Supplementary Table E

Summary of the individual radiologists' accuracy based on the reporting of the 'AI interpretable cases' only (n=259), along with the summary estimate based on the bivariate random effects meta-analysis, compared with the AI candidate accuracy across the same radiographs (i.e. Scenario 1).

Radiologist	Sensitivity	Specificity	PPV	NPV
**1**	81.2 (81.1 to 81.2)	87.7 (87.6 to 87.8)	88.2 (88.1 to 88.3)	80.5 (80.4 to 80.5)
**2**	67.6 (67.6 to 67.7)	91.7 (91.6 to 91.8)	90.4 (90.3 to 90.5)	71.2 (71.1 to 71.2)
**3**	85.4 (85.3 to 85.5)	81.3 (81.2 to 81.4)	83.6 (83.5 to 83.7)	83.3 (83.2 to 83.4)
**4**	90.0 (89.9 to 90.1)	87.5 (87.4 to 87.6)	89.4 (89.3 to 89.4)	88.2 (88.1 to 88.3)
**5**	85.0 (84.9 to 85.1)	89.2 (89.1 to 89.3)	90.2 (90.1 to 90.2)	83.6 (83.5 to 83.7)
**6**	77.9 (77.8 to 77.9)	87.6 (87.5 to 87.7)	86.4 (86.4 to 86.5)	79.6 (79.5 to 79.7)
**7**	88.2 (88.1 to 88.3)	83.1 (83.0 to 83.1)	85.1 (85.0 to 85.2)	86.6 (86.5 to 86.6)
**8**	73.1 (73.1 to 73.2)	79.4 (79.3 to 79.4)	79.0 (79.0 to 79.1)	73.5 (73.5 to 73.6)
**9**	84.9 (84.8 to 85.0)	89.3 (89.2 to 89.3)	90.1 (90.0 to 90.2)	83.7 (83.6 to 83.8)
**10**	89.9 (89.8 to 90.0)	86.8 (86.7 to 86.9)	88.7 (88.6 to 88.7)	88.2 (88.1 to 88.3)
**11**	91.8 (91.7 to 91.9)	77.8 (77.7 to 77.9)	81.5 (81.4 to 81.5)	89.9 (89.8 to 90.0)
**12**	77.9 (77.8 to 77.9)	94.2 (94.1 to 94.3)	94.0 (93.9 to 94.1)	78.5 (78.4 to 78.6)
**13**	65.2 (65.2 to 65.3)	94.1 (94.0 to 94.2)	92.9 (92.8 to 93.0)	69.6 (69.5 to 69.6)
**14**	91.1 (91.0 to 91.2)	79.2 (79.1 to 79.3)	82.6 (82.5 to 82.6)	89.2 (89.1 to 89.3)
**15**	79.6 (79.5 to 79.6)	87.0 (86.9 to 87.1)	87.2 (87.1 to 87.3)	79.3 (79.2 to 79.3)
**16**	82.0 (81.9 to 82.1)	81.0 (80.9 to 81.1)	83.2 (83.1 to 83.3)	79.7 (79.6 to 79.8)
**17**	92.8 (92.7 to 92.8)	86.1 (86.0 to 86.2)	88.3 (88.2 to 88.4)	91.3 (91.2 to 91.4)
**18**	87.9 (87.8 to 87.9)	95.8 (95.7 to 95.9)	96.1 (96.0 to 96.2)	87.1 (87.0 to 87.2)
**19**	75.2 (75.1 to 75.3)	87.8 (87.7 to 87.9)	87.3 (87.2 to 87.4)	76.1 (76.0 to 76.1)
**20**	82.0 (81.9 to 82.1)	92.6 (92.5 to 92.7)	92.7 (92.6 to 92.8)	81.8 (81.7 to 81.8)
**21**	85.6 (85.5 to 85.7)	86.0 (85.9 to 86.0)	87.5 (87.4 to 87.6)	83.9 (83.8 to 84.0)
**22**	89.6 (89.5 to 89.6)	75.4 (75.3 to 75.5)	79.5 (79.4 to 79.6)	87.2 (87.1 to 87.2)
**23**	70.7 (70.6 to 70.8)	89.2 (89.1 to 89.3)	88.4 (88.3 to 88.5)	72.3 (72.2 to 72.4)
**24**	83.0 (82.9 to 83.1)	95.0 (94.9 to 95.0)	95.1 (95.0 to 95.2)	82.5 (82.4 to 82.6)
**25**	97.0 (96.9 to 97.1)	83.2 (83.1 to 83.3)	86.2 (86.1 to 86.3)	96.3 (96.2 to 96.4)
**26**	89.4 (89.3 to 89.5)	94.9 (94.8 to 95.0)	95.5 (95.4 to 95.6)	88.2 (88.1 to 88.3)
**Summary Estimate***	84.1 (81.0- 87.0)	87.3 (85.0 - 89.3)		
**AI**	83.6 (76.2, 89.4)	75.2 (66.7, 82.5)	78.3 (72.5, 83.2)	81.0 (74.2, 86.4)

*Summary estimate from the bivariate random effects meta-analysis of radiologists performance

Supplementary Table F

Summary of the individual radiologists' accuracy based on the reporting of all radiographs (n=300) along with the summary estimate based on the bivariate random effects meta-analysis, compared with the AI accuracy across the same films (i.e. Scenario 4).

Radiologist	Sensitivity	Specificity	PPV	NPV
**1**	79.1 (79.0 to 79.2)	89.8 (89.7 to 89.9)	89.0 (88.9 to 89.1)	80.5 (80.4 to 80.6)
**2**	70.1 (70.1 to 70.2)	91.8 (91.7 to 91.9)	90.0 (89.9 to 90.1)	74.4 (74.4 to 74.5)
**3**	86.1 (86.0 to 86.2)	81.9 (81.8 to 82.0)	82.8 (82.7 to 82.9)	85.3 (85.2 to 85.4)
**4**	90.3 (90.2 to 90.4)	87.6 (87.5 to 87.7)	88.6 (88.5 to 88.7)	89.4 (89.3 to 89.5)
**5**	85.1 (85.0 to 85.1)	89.7 (89.6 to 89.8)	89.7 (89.6 to 89.8)	85.1 (85.0 to 85.1)
**6**	77.4 (77.3 to 77.5)	87.7 (87.6 to 87.7)	85.6 (85.5 to 85.7)	80.4 (80.3 to 80.4)
**7**	89.4 (89.3 to 89.5)	85.9 (85.8 to 86.0)	86.5 (86.5 to 86.6)	88.9 (88.8 to 89.0)
**8**	72.5 (72.4 to 72.6)	78.1 (78.1 to 78.2)	76.6 (76.5 to 76.7)	74.2 (74.1 to 74.3)
**9**	85.6 (85.5 to 85.7)	88.4 (88.3 to 88.5)	88.5 (88.4 to 88.6)	85.5 (85.4 to 85.6)
**10**	90.2 (90.1 to 90.3)	87.8 (87.7 to 87.8)	88.5 (88.4 to 88.5)	89.6 (89.5 to 89.7)
**11**	91.9 (91.8 to 92.0)	80.3 (80.2 to 80.3)	81.9 (81.8 to 82.0)	91.0 (91.0 to 91.1)
**12**	77.4 (77.3 to 77.5)	94.5 (94.4 to 94.6)	93.8 (93.7 to 93.8)	79.7 (79.6 to 79.7)
**13**	64.7 (64.7 to 64.8)	95.1 (95.0 to 95.2)	93.5 (93.4 to 93.6)	71.4 (71.3 to 71.4)
**14**	91.3 (91.2 to 91.4)	78.1 (78.1 to 78.2)	80.5 (80.4 to 80.6)	90.1 (90.0 to 90.2)
**15**	80.3 (80.2 to 80.3)	88.5 (88.4 to 88.6)	87.8 (87.7 to 87.9)	81.4 (81.3 to 81.4)
**16**	80.5 (80.4 to 80.6)	82.9 (82.8 to 83.0)	83.2 (83.1 to 83.3)	80.1 (80.0 to 80.2)
**17**	92.2 (92.1 to 92.2)	88.4 (88.3 to 88.5)	89.2 (89.2 to 89.3)	91.5 (91.5 to 91.6)
**18**	87.1 (87.0 to 87.2)	96.6 (96.5 to 96.6)	96.4 (96.3 to 96.5)	87.5 (87.4 to 87.6)
**19**	76.3 (76.2 to 76.4)	87.2 (87.1 to 87.2)	85.9 (85.8 to 86.0)	78.2 (78.1 to 78.3)
**20**	83.1 (83.0 to 83.2)	93.2 (93.1 to 93.2)	92.8 (92.7 to 92.8)	84.0 (83.9 to 84.0)
**21**	86.4 (86.3 to 86.4)	86.3 (86.2 to 86.4)	86.9 (86.8 to 87.0)	85.7 (85.6 to 85.8)
**22**	89.9 (89.8 to 90.0)	78.1 (78.1 to 78.2)	80.2 (80.2 to 80.3)	88.7 (88.6 to 88.8)
**23**	72.9 (72.8 to 73.0)	89.0 (88.9 to 89.1)	87.6 (87.5 to 87.7)	75.4 (75.4 to 75.5)
**24**	82.7 (82.6 to 82.8)	93.8 (93.7 to 93.8)	93.5 (93.4 to 93.6)	83.3 (83.2 to 83.4)
**25**	96.6 (96.6 to 96.7)	81.5 (81.4 to 81.5)	83.7 (83.6 to 83.8)	96.1 (96.0 to 96.2)
**26**	88.5 (88.4 to 88.6)	94.4 (94.3 to 94.5)	94.6 (94.5 to 94.6)	88.2 (88.1 to 88.3)
**Summary Estimate***	84.0 (80.8 to 86.7)	87.5 (84.8 to 89.8)		
**AI**	75.2 (67.4, 81.9)	62.3 (54.0, 70.0)	66.3 (61.1, 71.1)	71.8 (65.2, 77.5)

*Summary estimate from the bivariate random effects meta-analysis of radiologists performance
